# One Thousand Dollar Assist Heart Pump for Patients from Developing Countries

**DOI:** 10.2174/1874120700701010011

**Published:** 2007-06-28

**Authors:** Kun-xi Qian

**Affiliations:** Jiangsu University, Biomedical Engineering Institute, Zhenjiang, 212013, China

## Abstract

In spite of continuous improvements in device design and applications, the profound use of heart pump has been limited because of its high price. The available clinically applied heart pump costs mostly about 100 thousands US Dollars. The author has since long tried to develop a heart pump costing only 1000 Dollars for recovery or bridge to heart transplantation therapies. The device is a radially driven centrifugal pump with a brush-less DC motor and a streamlined impeller. Its bearing is rolling bearing using 4 to 6 needles, manufactured by special wear-proof polythene with super-high-molecular weight, thus the service life achieves more than 10 years. To avoid thrombus formation, a special purge system is introduced to the bearing, allowing the saline with heparin to be infused through the bearing into the pump. The bearing, therefore, keeps working in the saline, and absolutely no thrombus will be formed along the bearing. Animal experiments demonstrated that a 30 mL fluid infusion per hour is enough to prevent thrombus formation. With these improvements, the impeller pump has continuously run for 14 months in the laboratory, and no bearing wear can be measured. The device, weighing 150 g, is fully implantable, consumes approximately 9.6 W, delivers a 9Lmin-1 blood flow against a 120 mmHg mean pressure, and reaches a highest total efficiency of 24.7% for the motor (including the controller) and the pump. The device has been used in animal experiments together with an American artificial lung for more than one month in the University of Texas and also in human trials in the Taiwan University.

## INTRODUCTION

The R&D of artificial heart has over 50 years’ history. In the first 25 years, the diaphragm pumps imitating the function and mechanism of the natural heart had demonstrated the feasibility of man-made mechanical pumps in support or replacement of both left and right ventricles partly or totally. As soon as people realized that the diaphragm pump is too complicated, the simple rotary pump has been investigated extensively in the recent 25 years. Now an axial pump has worked on a patient for more than 7 years [1]. Sooner or later the heart pump will be an alternative to lacked natural donor heart in transplantation.

In spite of the technical progress in heart assist pump, its profound application has been limited because it costs about 100 k US Dollars. Most patients especially from the developing countries need a sufficient and reliable VAD but can not use such expensive device. The author has tried to develop a heart pump costing only 1000 dollars and has succeeded in the recent years [2]. This paper presents the structure and experimental results of the one thousand dollar heart pump and appeals for cooperation for further development.

## METHOD

Fig. (**1**) is a schematic drawing of the one thousand dollar blood pump. It consists of a motor and an impeller (2) in a pump housing (1). The motor has rotor magnets (3) and motor coil (4) winded on an iron core. The impeller is fixed on the rotor, which is bore by 4 to 6 needles (6) onto a stick screwed on the stator. The structure of the needle bearing is shown in Fig. (**2**). It’s well known that the rolling friction is much smaller than the sliding friction and that the needle material of polythene with super-high-molecular weight has excellent anti-wear property, the service life achieves thereafter over 10 years.

To prevent the blood penetrating into the gap between the rotor and the stator, a special purge system (5) is devised, allowing the saline with heparin to be infused through the bearing into the pump. The bearing, therefore, keeps working in the saline, and absolutely no thrombus will be formed. Animal experiments demonstrated that a 30 ml fluid infusion per hour is enough to prevent thrombus formation.

## RESULTS

**Fig. (4):**
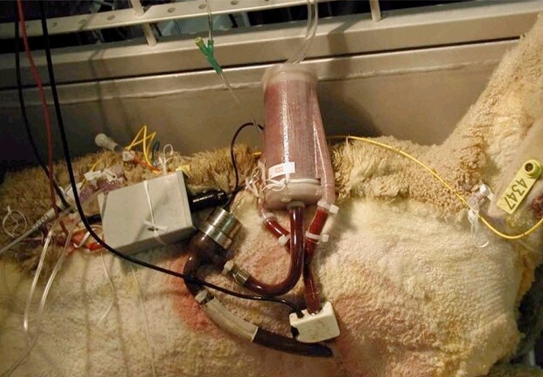
The pump with artificial lung in animal experiments. The survival achieved over 1 month.

Fig. (**3**) exhibits this elegant pump which had once continuously run for 14 months in the laboratory, and no bearing wear could be measured. The device, weighing 150 g, is fully implantable, consumes approximately 9.6 watts power, delivers a 9L/min blood flow against a 120 mm Hg mean pressure, and reaches a highest total efficiency of 24.7% for the motor (including the controller) and pump. The device has been used in animal experiments together with an American artificial lung for more than one month [3] in the University of Texas (Fig. **4**) and also in human trials in the Taiwan University [4].

## DISCUSSION

A simple, implantable, durable and reliable left ventricular assist device costing only one thousand US Dollars is presented, which may solve the present problems that hundreds and thousands patients, especially in developing countries, need a heart pump but cannot afford it due to its high costs. The author is ready to cooperate in further development of the device to be a clinically applicable device finally.

## Figures and Tables

**Fig. (1):**
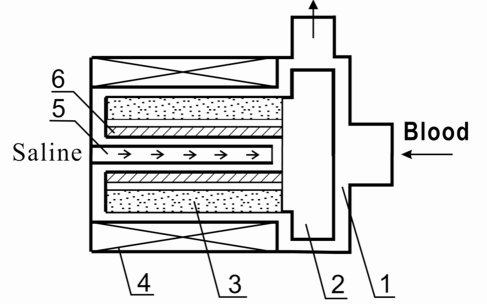
Radial driven centrifugal pump with needles bearing and purge system.

**Fig. (2):**
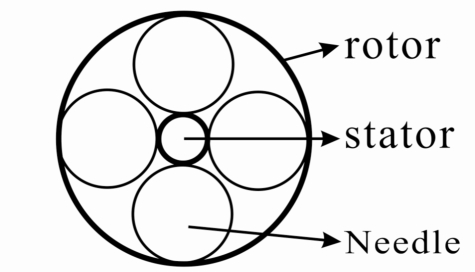
Needle bearing structure.

**Fig. (3):**
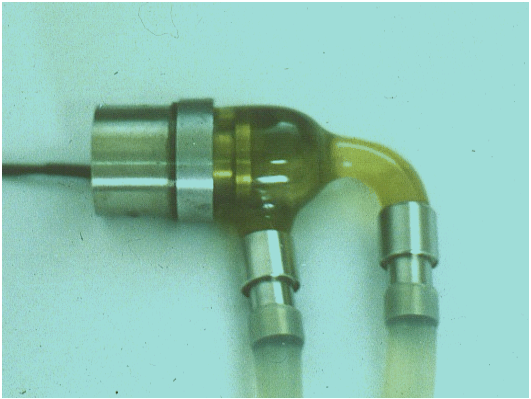
The elegant pump costs only one thousand dollars.
